# The Impact of Maximum Power Point Tracking Algorithms on Properties of On-Chip PV-Based Energy Harvester for IoT Devices †

**DOI:** 10.3390/s26031051

**Published:** 2026-02-05

**Authors:** Adam Hudec, Viera Stopjakova, Robert Ondica, Miroslav Potocny, Lukas Nagy

**Affiliations:** Department of IC Design and Test, Institute of Electronics and Photonics, Faculty of Electrical Engineering and Information Technology, Slovak University of Technology, Ilkovicova 3, 841 04 Bratislava, Slovakia; viera.stopjakova@stuba.sk (V.S.); robert.ondica@stuba.sk (R.O.); miroslav.potocny@stuba.sk (M.P.); lukas.nagy@stuba.sk (L.N.)

**Keywords:** energy harvester, MPPT algorithms, conversion efficiency, Perturb & Observe, constant voltage, pilot-cell

## Abstract

This article presents the analysis of selected maximum power point tracking (MPPT) algorithms and their influence on developed energy harvester (EH) systems under uniform conditions. The energy harvester is an electronic system that converts available ambient energy to electrical energy and regulates its distribution to the output. The aim is to design an energy harvester with the highest integration rate possible with consideration of area requirements and low power consumption. To improve the overall energy conversion of the developed harvester, we implemented several MPPT algorithms (Pilot Cell, Constant Voltage, Perturb and Observe) into a dedicated MPPT controller that controls the DC-DC converter. Consequently, we experimentally analyzed their impact on the harvester system. Findings show that even simple algorithms with smaller chip areas and lower power consumption can achieve results comparable to more complex ones. The proposed, manufactured and experimentally evaluated EH chip prototype has proven its expected functionality and is therefore fully capable of supplying energy for low-power electronics and battery-operated devices.

## 1. Introduction and Motivation

The research and development of electronics is mostly driven forward by actual needs of human society, but it is limited by materials and technologies of the given time. Materials and technologies are the dominant factors influencing the miniaturization of electronic systems or their level of integration on a chip. Reducing physical dimensions of components on a chip has a positive effect on the power consumption, while increasing the density of components brings more computing power [1]. These properties enable the emergence of smart and wireless electronics such as the Internet of Things (IoT) or wearable electronics.

Recently, we are approaching a point where providing enough computing power in a small area is no longer an issue for many applications. On the other hand, using energy sources more effectively and decreasing energy consumption are becoming more and more important. Wearable electronics, or IoT, make extensive use of various sensor systems for which a permanent source of electrical energy is unavailable and therefore, are powered by batteries. Battery-powered systems have a finite lifespan influenced by their own energy consumption and battery capacity. When the battery is discharged, it needs to be recharged or replaced. In the case of wearable electronics, this is of course not a problem, but in other applications like sensor systems in hard-to-reach places or implantable medical devices, such maintenance is either rather demanding or impossible. Therefore, it would be beneficial to avoid recharging/replacing batteries altogether.

Energy harvesters (EH) are the solution to the extension of the battery system lifespan [2]. EH is a system that harvests ambient energy from various sources (e.g., solar, thermal, mechanical, etc.) and converts it into electrical energy. High output power is often used in practice, employing energy harvesters that convert solar energy into electrical energy. Energy harvesting systems generally consist of an energy converter, a circuit that adapts the output voltage value (most often a DC-DC converter), and an energy storage that serves as the main energy source and a place where the excess converted energy is stored. Due to the nonlinear characteristics of photovoltaic (PV) cells or the inability to adapt to load changes, such a harvesting system is not able to function properly and independently or offer the maximum energy conversion efficiency, and therefore, the presence of a control circuit is necessary. In terms of simplicity, this can be either a Pulse-Width-Modulated (PWM) regulator that drives a power switch for energy distribution from the solar cells to the load or batteries. A more advanced option is to apply a DC-DC converter to provide more stable energy distribution using direct PWM/PFM control or an enhanced circuit providing the maximum power point tracking (MPPT).

There are several known algorithms that can tune the EH operating point, which approaches or overlaps the maximum power point (MPP). The MPP tuning should take as little time as possible to eliminate the MPP drift, eliminate deformations of the PV cell characteristics under partially shaded conditions, or adapt to material degradation due to overheating or aging. The solution to each of the above-mentioned problems increases the complexity of the MPPT algorithm, which leads to robustness of the respective digital circuit and an increase in the energy consumption and chip area overhead [3,4,5]. Digital circuits providing the MPPT control are generally less energy-hungry and occupy significantly less chip area compared to analog circuits, but despite these facts, their increase in total energy consumption can disable the whole energy harvester. Additionally, if they are very complex, the area overhead might be unacceptably large. Based on this information, one may conclude that when developing an energy harvester, it is necessary to take into account several parameters and make a compromise between them. This work therefore focuses on the analysis of selected MPPT algorithms and their impact on the entire energy harvester system in terms of several important aspects.

This paper is structured as follows: Section 1 brings motivation for the analysis of selected MPPT algorithms and their impact on the developed energy harvester. Section 2 describes energy harvesting systems for low-power electronics and offers a comparison of selected energy converters in terms of the main parameters. Section 3 presents typical types of energy regulation that can be implemented in solar energy harvesting systems. Section 4 provides an introduction into indirect and direct MPPT algorithms and summarizes the advantages and disadvantages of each. The general structure of the on-chip EH system (implemented in 65 nm CMOS technology) is described in Section 6, which also explains other off-chip components used to test the whole system. Evaluation of prototyped EH system chip samples using a robust measurement setup and achieved results are presented in Section 7. Discussion about the measured properties of each implemented MPPT algorithm within the EH system is given in Section 8. The Section 9 concludes the key findings and options for possible future improvements.

## 2. Energy Harvesters

Energy harvesters are not a newly emerging concept. On the contrary, they are well-known and widely utilized in the form of photovoltaic, hydro or wind power plants, which convert solar/mechanical energy into electrical energy, typically ranging from kilowatts to megawatts, and this capacity continues to grow.

As for devices more accessible for the general public, energy harvesters have appeared in products like calculators and LED lights. However, in recent years, they are increasingly found in more complex low-power electronics and systems-on-chip (SoC). Thus, for purposes of this paper, on-chip EH systems will be considered. As mentioned in Section 1, the enhancement of computational power and the reduction in energy consumption open doors for the development of sensor systems, wearable electronics and IoT devices. A critical component of such systems from an energy point of view is typically the power source, namely the battery. Its ability to supply the energy to a system is time-limited and depends on the value of the electrical load and ambient conditions. By utilizing energy harvesters, it is possible to extend the lifespan of battery-powered systems or, in special cases, even completely replace them.

A significant advantage of energy harvesters for low-power electronics is the possibility of applying other types of energy converters (ECs), such as piezoelectric (PZT), thermoelectric generators (TEGs), biofuel cells (BFCs) or radio frequency (RF) ones, in addition to those already mentioned. It is also useful to combine more converters to create so-called hybrid energy harvesting systems. These systems are more likely to be capable of supplying electrical energy for longer periods of time, or they may be able to provide power under conditions where a stand-alone converter would no longer have the necessary efficiency (such as a photovoltaic converter in the dark).

Energy harvesters are widely applicable systems, but the choice of the appropriate type of harvester depends on the specific application. When selecting parameters such as the final location as well as available surrounding energy sources that can be converted into electrical energy, the value of the voltage generated by the respective converter, its output power density, and last but not least, the physical dimensions must be considered.

In terms of the mentioned parameters, the most commonly used energy converter in EH systems is a solar cell. The output voltage of a standalone solar cell typically ranges in hundreds of millivolts, and by simply connecting multiple cells in series, one can achieve a voltage in the range of volts, which is suitable for many integrated circuits. The energy conversion efficiency is approximately 25%, and in some cases, it can reach up to 50% [6] or surpass this value [7]. Despite the relatively low efficiency, the available power density is in hundreds of $\mathrm{mW}/m^{2}$, which provides sufficient energy for many low-power electronic systems. A comparison of parameters of selected energy converters with the PV converter is presented in Table 1 [8,9,10,11,12,13,14,15,16].

## 3. PV Energy System Regulation

The most commonly converted energy using energy harvesters is solar energy, due to reasons and properties mentioned earlier. By connecting solar cells in series, one can achieve a higher output voltage, or by connecting them in parallel, a higher output current. Adjusting the output power from solar cells, whether on the voltage or current side, is a certain adaptation of the input conditions for the load. However, direct connection of PV cells to the load is inefficient and even dangerous, potentially leading to destructive effects. Thus, inserting a regulator between the solar cell and load can significantly prevent potential damage to the load. Furthermore, using a regulator might increase the efficiency of energy harvesting.

Just as the input conditions for the energy harvester change over time, so do the load conditions. Therefore, the role of a regulator is to adjust properties of the energy harvester to eliminate changes in conditions, whether at the input or output, to the greatest extent possible, for example, through pulse-width modulation (PWM) or more advanced methods of tracking the maximum power point of the EH system.

### 3.1. PWM Regulator

**Figure 1:**
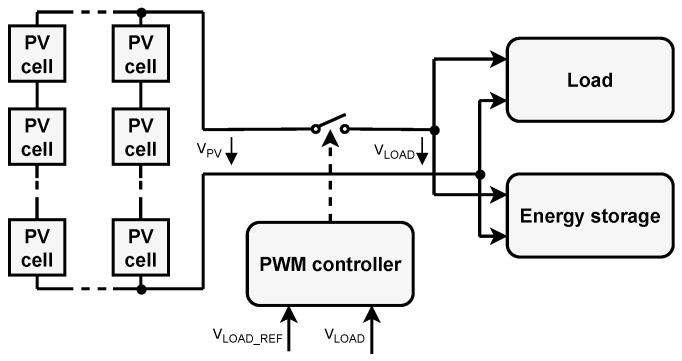
Block diagram of PV energy harvester with a PWM controller.

A straightforward method for controlling the distribution of energy from the harvester input to load or battery storage is based on direct connection/disconnection of PV cells to/from the load or energy storage through the controlled power switch [17,18] as it is depicted in Figure 1. In this case, the output voltage of the PV array is not regulated, but it is at least limited by the connection of solar cells.

It is typical for this type of regulator that the generated voltage on the solar cells must be equal to or close to the load reference voltage $V_{load\_ref}$ value. The PWM regulator typically operates as a solar battery charger; therefore, $V_{load\_ref}$ is set to the fully charged battery voltage. In order to generate the appropriate PWM control signal duty cycle, the reference load voltage $V_{load\_ref}$ and the instantaneous load voltage $V_{load}$, which serves as a feedback signal, must be present.

Under very high load or when the battery storage is depleted, the PWM controller (regulator) adjusts its output so that the current flowing to the load is unrestricted, meaning it is regulated solely by the load itself. As the value of $V_{load}$ increases, the current flowing into the load/battery is gradually limited until the condition $V_{load}>=V_{load\_ref}$ is met and the solar cells are disconnected from the rest of the circuit.

Simplicity of such a system positively impacts the cost, making it attractive for various applications. It can reliably transfer energy from the energy harvester input to its output and adapt to changing conditions at both the input and output of the energy harvester. During periods of excess solar energy, particularly in the summer, the system operates with high efficiency. At first glance, PWM regulation seems to be a suitable choice for any practical applications where cost is a primary concern. However, it is important to consider also the drawbacks of this regulation method. One of these is the number and configuration of solar cells necessary. Excessively high charging power with very depleted batteries can negatively affect the lifespan of the battery storage. Last but not least, the PWM regulator is unable to ensure a full charge of the energy storage or a sufficient voltage level for reliable operation of the powered system.

### 3.2. DC-DC Converter

An energy harvester with a PWM regulator adjusts the output power but cannot control the output voltage. This voltage depends on the number of solar cells, their interconnection, and the intensity of incoming sunlight but generally is limited mainly by connecting the solar cells in series.

**Figure 2:**
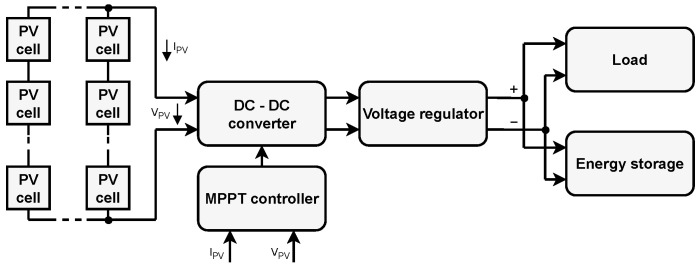
Block diagram of energy harvester with a MPPT controller.

To overcome the voltage limit imposed by the number of solar cells in series and the difference in irradiation conditions, it is necessary to insert a DC-DC converter between the energy harvester and the electrical load or energy storage [19]. For the DC-DC converter to operate optimally and efficiently, it must be controlled by an additional circuit, for example, a conventional PWM or PFM controller or a more elaborate MPPT controller. An example of the system with MPPT regulation is illustrated in Figure 2.

#### 3.2.1. PWM/PFM Control

DC-DC converters are commonly controlled by Pulse-Width-Modulated (PWM) or Pulse-Frequency-Modulated (PFM) signals. They secure a suitable output voltage by adjusting the duty cycle or frequency.

In the first case, the PWM is a technique for generating a signal with a constant period (T). The dynamic change in duty cycle (ratio of the duration of Log.1 ($T_{on}$) to the duration of one period) is obtained by change in $T_{on}$.

**Figure 3:**
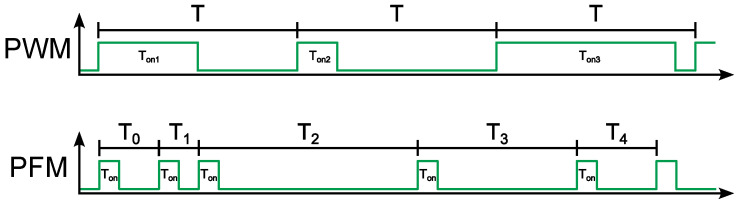
PWM and PFM signal waveforms.

In the second case, the PFM generates a signal with a fixed duration of Log.1 $\left(T_{on}\right)$ and a variable duration of Log.0. In this way, the frequency (period) of the control signal is adjusted. For illustration, Figure 3 displays the waveform of PWM and PFM signals.

A simple PWM (or PFM) controller commonly requires information about one regulated parameter, for example, the output voltage. Based on this measured parameter, the controller adjusts the switching phases of the converter, and the output voltage is regulated.

Obtaining and processing input data is one thing, but adapting the output voltage of the DC-DC converter based on this information is another. Such a voltage adaptation is achieved by generating a PFM or PWM control signal. In the case of PFM, the output voltage of the DC-DC converter is regulated by changing the frequency of the control signal. To achieve the highest possible voltage level, the higher frequency must be ensured. Nevertheless, high frequency negatively impacts power consumption of the system, increases switching losses and electromagnetic interference, and consequently, decreases the overall efficiency.

The second option is PWM regulation, which operates with a constant control signal frequency, and the duty cycle changes. Since the switching frequency is constant, it is accompanied by constant losses, which creates an imaginary boundary between PWM and PFM to choose the right control signal modulation method.

Since constant switching frequency means constant losses which, when small in relation to the transmitted power, makes PWM control more efficient than PFM. On the other hand, if the value of the transmitted power is small, while switching at constant frequency, switching losses can exceed a certain value and this control becomes inefficient.

**Figure 4:**
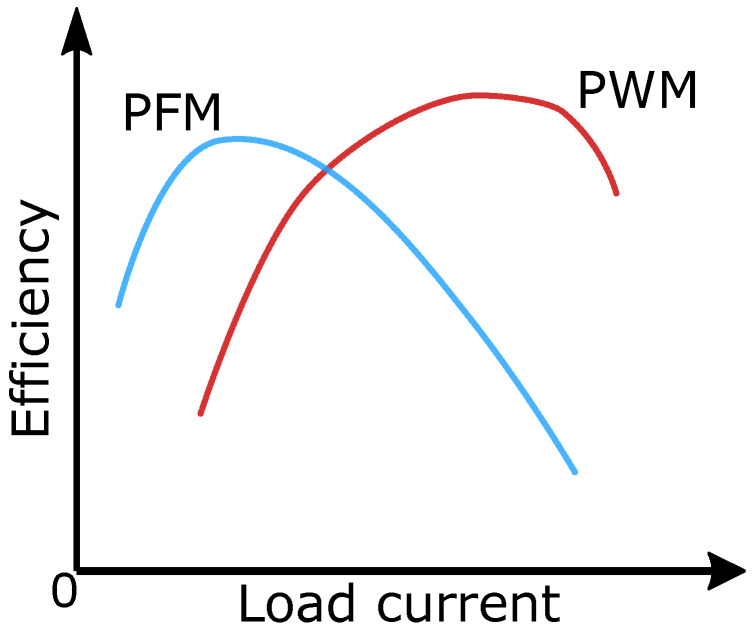
Power efficiency of PFM and PWM vs load current.

The general power ranges and efficiency levels for which the selected type of modulation of the control signal is suitable are shown in Figure 4 [20,21,22]. In our work, we chose the right PFM, as the goal is to work with low power.

#### 3.2.2. MPPT Control

**Figure 5:**
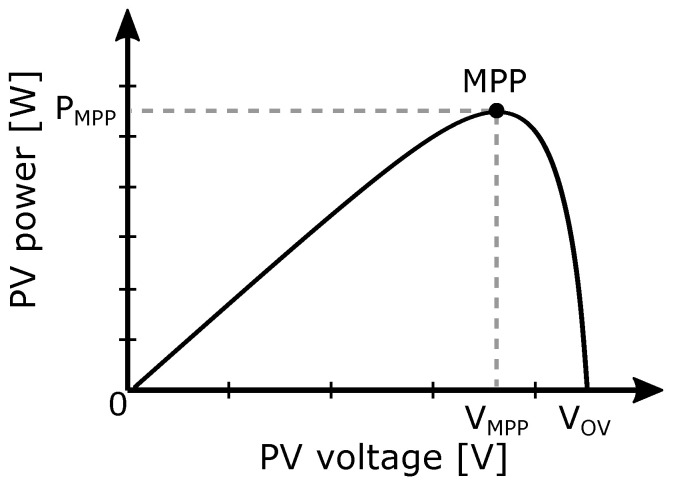
P-V curve of a solar cell and MPP tracking.

The main role of the MPPT regulator is to evaluate the measured input parameters, specifically voltage and current, and to adjust or shift the operating point on the power–voltage (P-V) curve of the solar cell (Figure 5) to the maximum power point that can be obtained under given conditions, thereby maximizing energy extraction and the efficiency of the energy harvester.

In regulating the output power of the energy harvester, a wide range of algorithms exist, each suitable for a specific application. Simpler algorithms only need to measure one of the input parameters, voltage or current, and achieve high tuning speeds of the operating point, albeit at the cost of never reaching the actual $V_{mpp}$ value. More complex algorithms for precise regulation and achieving higher efficiency require providing both variables, and it is possible to expand the inputs to include information about the intensity of ambient light irradiation or temperature.

## 4. MPPT Algorithms

The most commonly used energy converter is the solar cell due to its relatively high output voltage, provided power density and stability of the output power. Its current-voltage (I-V) characteristic, or power–voltage (P-V) relationship, is non-linear and typically contains only one maximum, as shown in Figure 5. When the operating point is located at the MPP on the P-V curve, we say that the system is tuned for the maximum energy extraction. The position of the operating point is determined by parameters such as the amount of incoming radiation on PV cells or the size of loads. Since these conditions change dynamically, the position of the operating point shifts uncontrollably and may lead to inefficient energy extraction and potentially disable the entire system. To control and set the operating point to the MPP position, the aforementioned PWM regulator is commonly used, due to its simplicity. To obtain higher power conversion efficiency and higher accuracy, the more advanced regulator based on MPPT algorithms [23] can be implemented. Based on the type of inputs and the method of tuning the operating point, MPPT algorithms can be categorized into two groups: indirect and direct algorithms.

### 4.1. Indirect Algorithms

Indirect MPPT algorithms can be described as algorithms that primarily work with static variables. They process information from a general model or data obtained from pre-application characterization of the PV cell. This involves laboratory measurements acquired from the I-V and P-V curves in an unloaded state, from which we can extract important parameters such as open-circuit voltage $V_{OV}$, short-circuit current $I_{SC}$, and voltage $V_{MPP}$ and current $I_{MPP}$ at the MPP.

Based on the obtained data, it is possible to achieve the proper setting of the operating point of the energy converter for the maximum energy extraction. However, it is not possible to dynamically respond to changes in input conditions caused by factors such as changes in illumination, partial shading of the PV cell, and last but not least, material degradation due to aging. Individual indirect algorithms, by their very nature, will never reach the actual MPP but will approach and oscillate in its vicinity.

Despite the aforementioned drawbacks of indirect algorithms, some of them are very interesting for many applications. For example, algorithms like Constant Voltage (CV) [24], Pilot-cell (PC), Fractional Open-Circuit Voltage (FOCV), Fractional Short-Circuit Current (FSCC) [25], and others dominate in speed of tuning the operating point and are hardware-simple, which means they are not demanding in terms of area size and electrical energy consumption.

### 4.2. Direct Algorithms

Direct MPPT algorithms continuously adjust the operational point of the energy harvester based on real-time measurements of voltage $V_{PV}$ and current $I_{PV}$, and thus power $P_{PV}$, using an analog-to-digital converter. They do not require disconnecting the solar cell from the rest of the circuit, as is the case with some indirect algorithms.

Additionally, in contrast to indirect MPPT algorithms, direct MPPT algorithms do not require absolute knowledge of the I-V and P-V curves of the solar cell. Instead, they can dynamically adapt to changes in input conditions such as lighting, partial shading of the PV panel, temperature, and even material degradation.

The operation of direct algorithms using relative values—specifically by comparing current and previous measurements—enables the energy harvester to function effectively across a wide range of conditions while maintaining the highest possible extraction efficiency.

Characteristics such as high accuracy in tuning the operational point, responsiveness to dynamic changes, and high efficiency are typical features of even the simplest direct algorithms, such as Perturb and Observe (P&O) [26] and Incremental Conductance (IncCond) [27]. Of course, there are also many other algorithms, particularly those based on fuzzy logic [28] or artificial intelligence [29], which can better manage local maximums on the P-V curve generated by partially shaded PV panels or can eliminate MPPT drift [30]. However, such algorithms may require significantly more complex hardware and higher computational power.

A comparison of the properties of indirect and direct algorithms operating under uniform conditions is presented in Table 2 [31,32].

## 5. Implemented MPPT Algorithms

The proposed on-chip energy harvesting system for low-power electronics, incorporating a fully integrated DC–DC converter including the inductor, represents the first iteration of the presented research and development. Due to the complexity and robustness of the overall system, full-system simulations are not feasible, as they are computationally intensive and would require several months to complete even for a single configuration.

Since power consumption of the whole system could not be obtained through simulation, and an MPPT algorithm was required for the control of the DC–DC converter, it was assumed that digital control circuitry must exhibit the minimum power consumption (as the available power margin was unknown). Generally, the power consumption of digital circuits can be expressed by Equation (1).

(1) $$P_{total}=P_{static}+P_{dynamic}$$

The static power component $P_{static}$ corresponds to supply voltage ($V_{dd}$) and leakage current ($I_{leak}$), as shown in Equation (2) [33]), and its value mainly depends on the number of logic gates and flip-flops employed.

(2) $$P_{static}=V_{dd}+I_{leak}$$

The dynamic power $P_{dynamic}$ is defined by Equation (3) [34], with the supply voltage $V_{dd}$ being the dominant contributor, as dynamic power consumption scales quadratically with $V_{dd}$.

(3) $$P_{dynamic}=\alpha C_{L}fV_{dd}^{2},$$

where α is activity factor, $C_{L}$ represents the load capacitance, *f* is the clock frequency and $V_{dd}^{2}$ is the power supply voltage. Clock frequency *f* and load capacitance $C_{L}$ may also significantly affect the power consumption; however, these parameters are determined by circuit complexity. Within the dynamic power component, the activity factor α has the most significant impact, as it represents the fraction of digital nodes switching from logic zero to logic one.

To ensure continuous power delivery to the energy harvesting system and based on the considerations discussed above, indirect MPPT algorithms, such as Pilot-Cell and Constant Voltage, were selected for implementation in the developed harvesting system. Among the direct MPPT methods, the Perturb and Observe algorithm was adopted.

### 5.1. Pilot-Cell

The Pilot-Cell (PC) algorithm is based on Fractional Open-Circuit Voltage (FOCV) or Fractional Short-Circuit Current (FSCC) methods. The main drawback of both approaches is that FOCV requires a brief disconnection of the solar cell from the rest of the circuit, while FSCC involves temporarily short-circuiting the solar cell. These operations are necessary to obtain the open-circuit voltage or the short-circuit current of the solar cell, respectively. Using these values, the maximum power point voltage $V_{mpp}$ or current $I_{mpp}$ can be estimated according to Equations (4) and (5).

(4) $$V_{mpp}=kV_{ov}$$

(5) $$I_{mpp}=kI_{sc}$$

In both expressions, proportionality coefficient *k* appears, which depends on the solar cell type and illumination conditions. Its value typically ranges from 0.72 to 0.78 for FOCV and from 0.72 to 0.92 for FSCC [35].

During the aforementioned short interruptions of the energy harvesting process, power losses occur in the best-case scenario, while in the worst case, the energy harvester may shut down and fail to restart. These issues are effectively eliminated by the Pilot-Cell algorithm. Conceptually, the PC method extracts information from two solar cells placed in close proximity and exhibiting identical electrical characteristics. One solar cell serves as the primary energy source and is continuously loaded, providing $V_{pv}$ voltage and $I_{pv}$ current values, while the second solar cell acts as a reference, providing $V_{mpp}$ and $I_{mpp}$.

**Figure 6:**
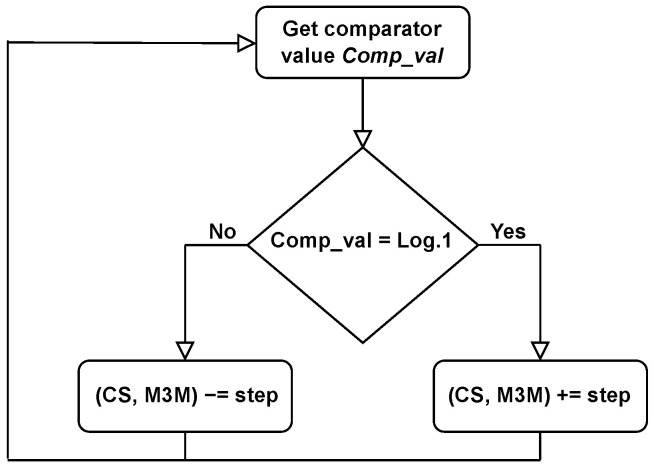
Flow diagram of Pilot-Cell algorithm.

In the proposed system, the illumination conditions are assumed to be static; therefore, the reference values remain constant. Furthermore, the PC algorithm does not directly process the absolute values of $V_{mpp}$ and $V_{pv}$, but instead operates with a 1-bit signal provided by a preceding comparator, as illustrated in Figure 6.

### 5.2. Constant Voltage

The Constant Voltage (CV) algorithm is the simplest MPPT technique. The CV method assumes only minor variations in irradiance and ambient temperature; therefore, the reference voltage can be considered constant throughout the operation. The main advantage of this algorithm is that it requires only a single solar cell for proper operation, and the reference value $V_{mpp}$ at a given illumination level is treated as a constant, which is precomputed according to Equation (4).

**Figure 7:**
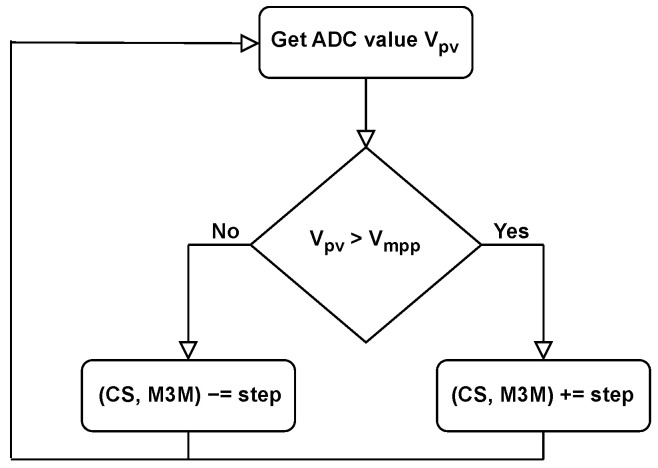
Flow diagram of Constant Voltage algorithm.

The on-chip implemented algorithm operates with a predefined value of $V_{mpp}$ corresponding to the given illumination conditions, which is stored in a register memory. This value was calculated using Equation (4), with the proportionality coefficient *k* selected in the range from 0.72 to 0.78. The voltage of the loaded solar cell $V_{pv}$ is obtained from the Analog-to-Digital Converter (ADC) and compared with the stored $V_{mpp}$, as depicted in Figure 7.

Both PC and CV algorithms exhibit a similarly simple control-flow structure; therefore, comparable characteristics in terms of power consumption and silicon area are expected. The convergence speed of the CV algorithm may be lower, since the ADC conversion time is longer than the comparator evaluation time. The steady-state operating-point error relative to the MPP is expected to be comparable to that of the PC algorithm. A larger deviation may occur only due to the limited resolution of the ADC or an inaccurately calculated digital representation of $V_{mpp}$. To achieve optimum energy extraction performance for both algorithms, the employed solar cell would need to be characterized in advance to determine the exact value of $V_{mpp}$ under specific illumination conditions.

### 5.3. Perturb and Observe

The Perturb and Observe algorithm is considered the simplest and generally most effective method among direct MPPT techniques. It does not rely on prior characterization or predefined constants; instead, it operates directly on the measured input voltage $V_{pv}$ and input current $I_{pv}$ obtained from the ADC. This direct measurement approach enables reliable operation under dynamically varying illumination conditions and eliminates the need for a reference or target voltage $V_{mpp}$. The algorithm intentionally perturbs the operating point through adjustments of the control signal generated for the DC–DC converter and subsequently evaluates the resulting change, thereby determining the direction toward the maximum power point.

**Figure 8:**
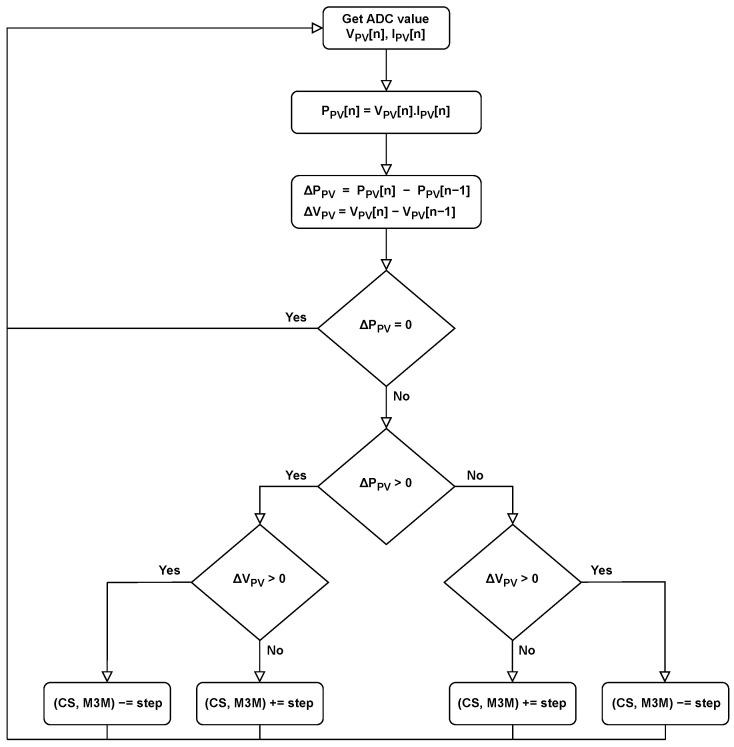
Flow diagram of Perturb and Observe algorithm.

The implemented on-chip P&O algorithm follows the standard formulation without additional modifications. Initially, the current values of $V_{pv}$ and $I_{pv}$ are acquired from the ADC. Since the primary decision mechanism is based on monitoring the change in input power, these two quantities are multiplied to obtain the instantaneous input power. In the subsequent steps, the newly acquired values are compared to those obtained in the previous iteration. Based on this evaluation, the frequency of the control signal is adjusted, which shifts the operating point along the P–V characteristic of the solar cell. The principle of the P&O algorithm is illustrated in Figure 8.

The P&O MPPT algorithm does not require a pilot or reference solar cell, nor does it involve disconnecting the solar cell from the system. It operates solely on measured data and does not depend on predefined characteristics or constants which, when combined with an ADC of sufficient resolution, results in a favorable steady-state error with respect to the MPP.

On the other hand, similarly to the CV algorithm discussed above, the convergence toward the MPP is slower compared to the PC algorithm, as the ADC conversion requires several clock cycles. In this case, the convergence delay is further increased by the multiplication of the input quantities, which also consumes multiple clock cycles. Consequently, the P&O algorithm exhibits the slowest convergence among the considered methods.

The implemented multiplier is not a simple digital circuit. Its complexity imposes significantly higher demands on silicon area, which is inherently associated with increased power consumption.

## 6. Developed On-Chip Energy Harvester

**Figure 9:**
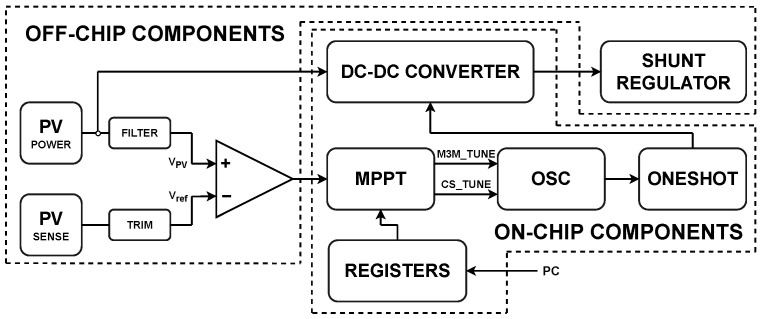
Simplified block diagram of the proposed on-chip energy harvester.

We have designed an energy harvester that converts solar energy into electrical energy. This is a relatively complex microelectronic system, where the goal was to achieve the highest possible level of integration on a chip using standard 65 nm CMOS technology. Its simplified block diagram is depicted in Figure 9. This figure illustrates parts that have been integrated onto the chip and also discrete components used externally.

### 6.1. On-Chip Components

Parts of the EH system integrated on a chip are the following.

**Figure 10:**
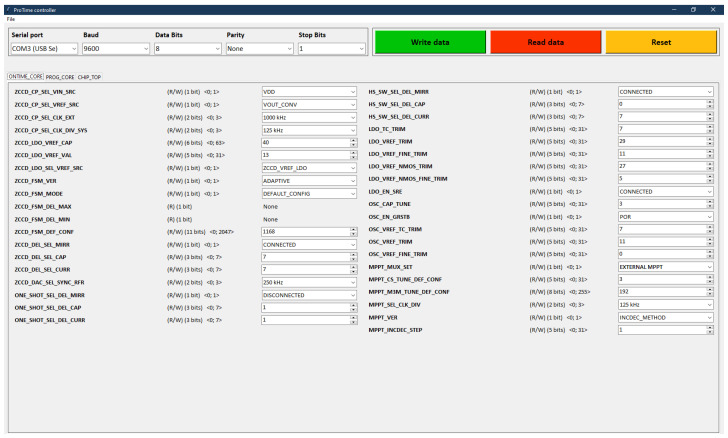
Graphical User Interface for manipulating the registers content.

- **DC-DC Converter**: Proposed voltage converter specifically for operation with a custom designed and fully integrated inductor on a chip [36,37,38,39] is based on the *Conventional Boost Converter (CBC)* topology [40,41,42]. The CBC topology was selected based on characteristics of the proposed system. The main requirement was to generate a stable 1.5 V output from two series-connected PV cells, which under ideal conditions (MPP) produce approximately 0.56 V. This necessitates the use of a step-up (boost) voltage converter. Consequently, the first prototype of the EH system was implemented using the simplest possible topology, with the goal of minimizing power dissipation by reducing the number of power devices, and for simplicity and reduced power consumption of required additional control circuitry. Its main components, such as power switches, inductors and parts of the input capacitor, are fully integrated on the chip. The low-side power switch is controlled by a PFM signal that was generated by a control loop. This high-side power switch is driven as an ideal diode with zero threshold voltage by a developed *Zero Current Crossing Detector (ZCCD)* [43] that is a part of the converter.
- **Control Loop**: The control loop adjusts the input impedance of the DC-DC converter and, in this way, effectively shifts the EH operating point to a position that ensures the maximum energy extraction from the input under given conditions. This energy is transferred to the output of the energy harvester. Main components of the control loop are fully integrated on a chip. The core of the control loop consists of the following circuits:
  **–**  **Registers**: The energy harvester is a complex electronic system integrated on a chip with targeted parameters. During the IC manufacturing, fluctuation of process occurs that may affect the EH system parameters. For this reason, we have added the capability for certain corrections/modifications of parameters using compensation banks, tuning banks and switches that can be controlled externally through register memory, i.e., via a computer. In the case of the MPPT block, it is possible to adjust the initialization parameters, change the type of internal MPPT algorithm or activate the option of connecting an external MPPT circuit implemented in a *Field Programmable Gate Array (FPGA)* form. The energy harvester contains a relatively large number of registers. For better control and interaction with registers during measurement, a custom *Graphical User Interface (GUI)* was developed in Python 3.9 programming language using the Tkinter tool. The developed GUI is shown in Figure 10.
  **–**  **MPPT**: The regulation of energy transfer from the energy converter input to its output is managed by a control signal, which can be of the PWM type or PFM type. Since the entire system was designed for low-power applications, we implemented a circuit for generating a PFM signal. The generation of the proper frequency for the control signal is determined by a circuit operating on the MPPT algorithm. Depending on the algorithm type, various input information is evaluated, such as a 1-bit comparison result of voltages on the loaded and reference solar cells for *Pilot-Cell (PC)* algorithm or in the case of *Constant Voltage (CV)* and *Perturb & Observe (P&O)* algorithms, it involves 10-bit numbers from ADC (Analog-to-Digital Converter) representing the voltage or current on the loaded solar cell. The Pilot-Cell algorithm with both adaptive step size and constant step size (adjustable via tuning registers ranging from 1 to 31) has been implemented on the chip. The output of the MPPT block is not directly the PFM control signal but rather data in two registers labeled CS and M3M, which serve as the input data for the oscillator block (OSC).
  **–**  **OSC**: A dynamically tunable relaxation oscillator is a part of the regulation loop, and its output signal frequency can be adjusted using a bank of capacitors and bank of current sources. The connection and disconnection of individual components in the banks are determined by a combination of data that the MPPT block provides to the oscillator from its own registers *CS* and *M3M*.
  **–**  **One-Shot**: Rectangular signal with 50% duty cycle generated by relaxation oscillator excites One-Shot oscillator that generates a pulse with defined width. For testing purposes, this pulse width can be further adjusted by Registers block.

### 6.2. Off-Chip Components

- **Solar Cells**: The proposed energy harvester converts solar energy into electrical energy using a solar cell. Our goal was to design and fully integrate a DC-DC converter with its entire control loop on a chip. For this reason, we applied *IXOLAR KXOB25-14X1TF* solar cell, which was selected based on parameters such as physical dimensions and the output power. The power that the solar cell can deliver at its output is sufficient, but the voltage value is low. Based on this fact, we added another solar cell in series, achieving an adequate voltage level for the system operation. Some MPPT algorithms, such as *Open Circuit Voltage* or *Short Circuit Current*, require temporarily interrupting the supply of extracted energy to the rest of the energy harvester circuit at regular intervals for proper functioning. This functionality updates the position of the actual MPP based on current lighting conditions. To ensure that such an interruption does not occur while also allowing space for the future application of the mentioned MPPT algorithms, we placed another identical assembly of solar cells next to the existing one. In this configuration, one PV cell pair serves as the source of extracted energy, while the other pair provides the reference voltage.
- **Measuring Circuits**: The proposed EH integrated circuit uses the Pilot-Cell MPPT algorithm. Decision-making occurs based on information from a voltage comparator that compares the voltage $V_{PV}$ of a loaded solar cell pair to the reference pair voltage (representing the $V_{MPP}$ value). To test other MPPT algorithms that can be implemented in FPGA and connected to the rest of the energy harvester, it is possible to eliminate the reference pair of solar cells and replace the voltage comparator with an ADC.
- **Shunt Regulator**: The energy harvester with a feedback-free control loop aims to maximize energy extraction at the input and transfer this power to its output, resulting in an output voltage $V_{OUT}$ from the energy harvester that is not regulated. To stabilize the output voltage level, we implemented a Shunt Regulator into the DC-DC converter, which maintains the output voltage value of 1.5 V.

### 6.3. Layout of the Proposed Energy Harvester

**Figure 11:**
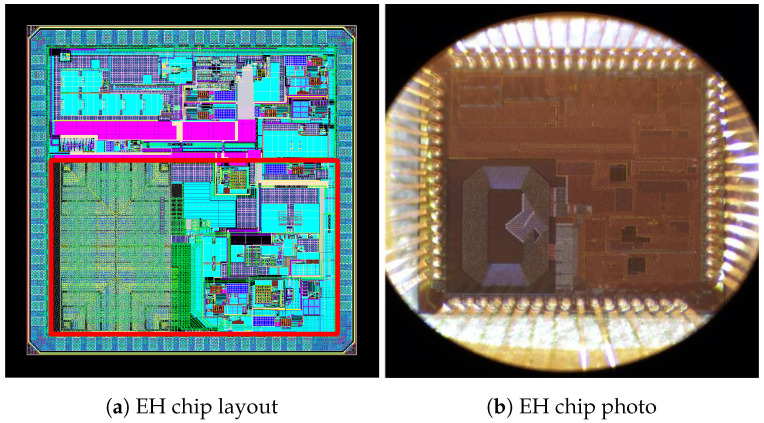
View of the EH layout and a photo of the manufactured chip.

The developed EH chip consists of several circuits described above (as blocks). Despite the complexity and robustness, the overall system physical dimensions are only 1.6 mm × 1 mm, and layout of the EH chip is depicted in Figure 11a (highlighted by a red rectangle), with the most recognizable part being the inductor located at the bottom left.

The chip itself was manufactured in 65nm CMOS technology by UMC, with a volume of 50 samples being manufactured, of which 10 were encapsulated. In Figure 11b, a micro-photograph of the packaged chip is shown. To evaluate and characterize the EH system and, particularly, the implemented MPPT algorithms, rather comprehensive experimental measurements were performed on the prototyped samples.

## 7. Measurement Results

**Figure 12:**
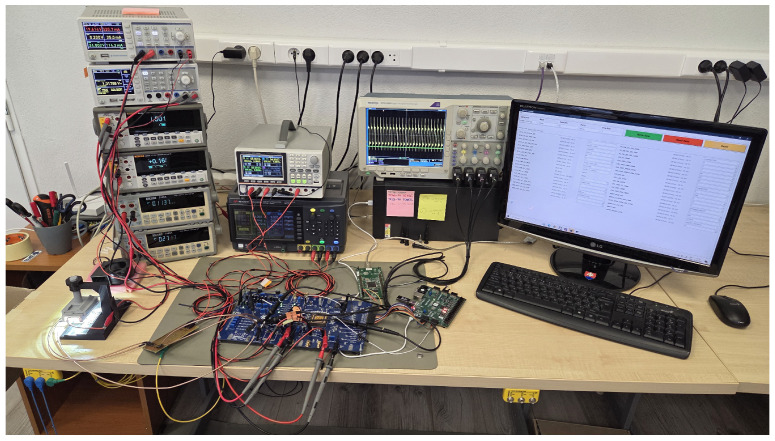
Measurement setup.

To evaluate such a complex EH system through measurement of its parameters and properties, it was necessary to assemble a measurement setup shown in Figure 12. For accurate control of the PV cell irradiation and prevention of the interference from ambient lighting, a protective chamber was additionally put over the solar cells during the measurement. Due to the need to measure a relatively large number of EH chip parameters, it was essential to use multiple measurement instruments such as the following:

- **Computer**: It is used to modify the content of control registers through a developed GUI and to store and evaluate selected measured parameters of the entire system.
- **FPGA Board (NEXYS A7-100T)**: The FPGA board is connected to the EH chip and to the computer through a test board and *Universal Serial Bus (USB)* interface, respectively. Firstly, the basic functionality of the on-chip implemented MPPT algorithms was evaluated and proved by measurements. Using the FPGA board, the exact copy of the aforementioned MPPT algorithms was also externally implemented into the EH control loop for better control of the full measurement process and better reading of the state of tuning registers. The functionality of the used FPGA board is not limited to the implementation of individual MPPT algorithms; the board also serves as a readout device for information (such as voltage and current at the energy harvester input) obtained through an ADC and the combination of CS and M3M registers that tune the relaxation oscillator frequency.
- **Oscilloscope (TEKTRONIX DPO4104B)**: The oscilloscope is connected to the test board, which features an output for sensing the frequency $f_{SW}$ of the dynamically tunable relaxation oscillator.
- **Power Supplies (KEYSIGHT EDU36311A, GW-INSTEK GPP-4323, R&S HMC8043)**: The individual power supply sources serve to power the chip test board, the additional matching circuit with a two-channel ADC, and the illumination of the solar cells.
- **Multimeters (FLUKE 8845A, R&S HMC8012, ESCORT 3146A)**:Laboratory multimeters were used to measure the output voltage $V_{OUT}$, the output current $I_{OUT}$ flowing through the Shunt Regulator, the voltage value of the on-chip voltage regulator $V_{LDO}$, and currents flowing through the main power branches.

Before measurements of the energy harvester as a complete system, we first conducted several preliminary measurements. The aim was to obtain the radiation characteristics from the employed light source, characteristics of the solar cells connected in series under various lighting conditions, and to determine the minimum and maximum light levels at which the energy harvester still operates properly. Our measurements revealed that the system operation is stable within the radiation range from $425\;W/m^{2}$ to $500\;W/m^{2}$. Thus, these extreme radiation values were applied in subsequent measurements. We gradually applied individual MPPT algorithms (i.e. PC, P&O, CV) to the EH control loop. For the PC algorithm, which processes 1-bit information from the voltage comparator, it was possible to adjust the step size down to 1. In the case of the P&O and CV algorithms, the step size could not be lower than 15, which is due to the inadequately chosen resolution of the employed ADC. To objectively compare the behavior of the energy harvester when implementing various MPPT algorithms, the tuning step for all algorithms was set to values of 15 and 31.

**Figure 13:**
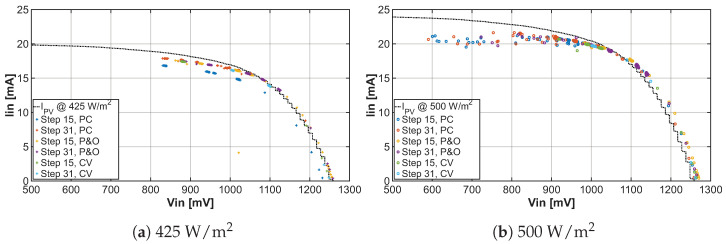
Comparison I-V characteristics under light irradiance $425\;W/m^{2}$ and $500\;W/m^{2}$ for different MPPT algorithms with tuning steps 15 and 31.

**Figure 14:**
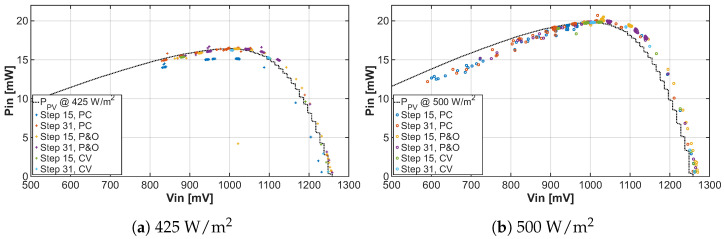
Comparison P-V characteristic under light irradiance $425\;W/m^{2}$ and $500\;W/m^{2}$ for different MPPT algorithms with tuning steps 15 and 31.

Since the goal of MPPT algorithms is to tune the operating point to the maximum power point, we recorded the individual positions of the operating point during the tuning process and plotted them in graphs representing the I-V characteristic and P-V characteristic shown in Figure 13 and Figure 14 of the solar cell under two different lighting conditions, respectively. After adding the real characteristics of solar cells to the graphs, one can observe that the implemented MPPT algorithms correctly seek the maximum power point and even reach it with a certain deviation.

**Figure 15:**
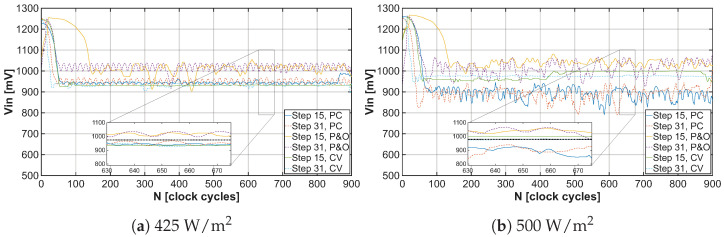
Convergence of $V_{IN}$ to $V_{MPP}$ under light irradiance $425\;W/m^{2}$ and $500\;W/m^{2}$ for different MPPT algorithms with tuning steps 15 and 31.

**Figure 16:**
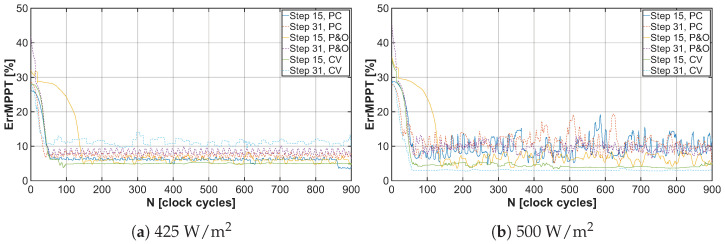
Achieved MPP mismatch from real MPP under light irradiance $425\;W/m^{2}$ and $500\;W/m^{2}$ for different MPPT algorithms using tuning steps 15 and 31.

The tuning process of the operating point begins at the open-circuit voltage $V_{OV}$ of the solar cell. By gradually adjusting the frequency of the control signal for the DC-DC converter using the MPPT algorithm, the system converges to the $V_{MPP}$ value, as represented in the results shown in Figure 15a,b, each N value meaning one step of the convergence process. The magnitude of deviation from the actual $V_{MPP}$ value of the solar cell is represented by the curves depicted in Figure 16a,b.

**Figure 17:**
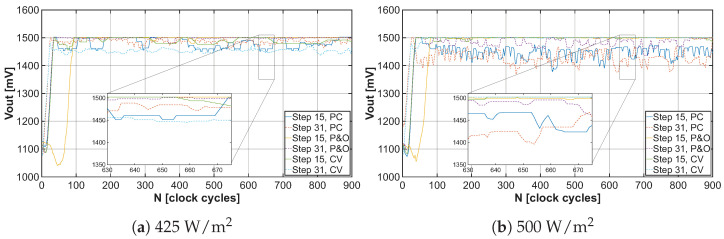
Output voltage stability under light irradiance $425\;W/m^{2}$ and $500\;W/m^{2}$ for different MPPT algorithms with tuning steps 15 and 31.

**Figure 18:**
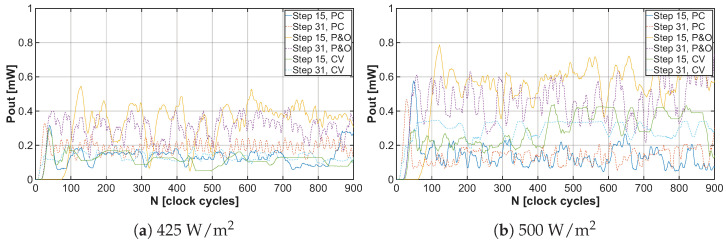
Amount of available output power under light irradiance $425\;W/m^{2}$ and $500\;W/m^{2}$ for different MPPT algorithms with tuning steps 15 and 31.

As already mentioned, the energy harvester with MPPT control aims to extract the maximum power from the solar cells, which in practice means that the DC-DC converter increases the output voltage $V_{OUT}$ but the shunt regulator limits it to the value of 1.5V, as can be observed in Figure 17a,b. When the output voltage reaches this specified value, the energy converter has enough energy for its proper functioning, and the excess energy can be utilized for supplying the load or battery systems, as shown in Figure 18a,b.

**Figure 19:**
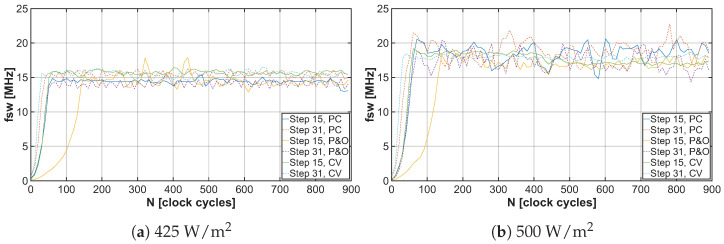
Relaxation oscillator frequency under light irradiance $425\;W/m^{2}$ and $500\;W/m^{2}$ for different MPPT algorithms with tuning steps 15 and 31.

**Figure 20:**
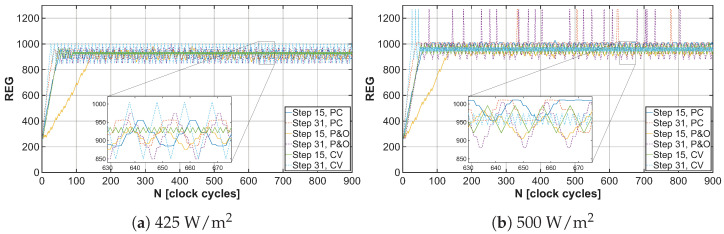
Speed of convergence and oscillations in MPP under light irradiance $425\;W/m^{2}$ and $500\;W/m^{2}$ for different MPPT algorithms with tuning steps 15 and 31.

The developed energy harvester for low-power applications is controlled using a PFM signal, which drives the low-side switch of the DC-DC converter, thereby adjusting the input impedance. The control signal frequency is determined by the MPPT algorithm and generated by the relaxation oscillator. The variation in the PFM signal frequency based on captured samples can be observed in Figure 19a,b. Based on these graphs, it is possible to determine how long it takes for each MPPT algorithm to tune the EH system to the maximum power point. Since the frequency of the PFM signal at each point in the graphs is represented by a combination of data contained in the CS and M3M registers, serializing them allows for a simpler determination of how many clock cycles it takes for each MPPT algorithm to reach the MPP. These trends can be observed in Figure 20a,b.

## 8. Discussion

The proposed energy harvesting system implemented in 65 nm CMOS technology has been proven functional by measurement of prototyped chip samples. Since the option for external FPGA connection within the chip was implemented, we were able to incorporate both the MPPT algorithms (P&O and CV). This allowed us to subsequently inspect the entire system and gradually capture data such as the current configuration of CS and M3M registers, oscillator frequency, the input and output power, convergence speed to the MPP, and other derived parameters. We analyzed the acquired data, focusing particularly on the most influenced by the applied MPPT algorithms.

In Table 3 and Table 4, we present four important parameters of the developed EH system. The first of all is the convergence speed to the maximum power point. The change in input conditions of solar energy harvesters is usually smooth and long-lasting, but in IoT or other wearable electronics, this change can occur very fast. It is very important that the energy harvester can adapt to this change as quickly as possible and maintain the best possible performance. The energy harvester must be able to respond not only to changes in input conditions but also to manage output conditions that are determined by the load behavior. If there is no rapid response to changes in input or load conditions, excessive energy may be drawn from the batteries (even when batteries are not in use), or the entire system may fail if the batteries are discharged.

The second parameter is the voltage error at the input compared to the actual voltage at the MPP. The smaller this error is, the more energy the energy harvester will be able to extract from the input and create a larger power margin for the load, or it will be able to supply excess energy to the battery storage.

The third analyzed parameter is the MPPT power consumption (dynamic and static). This is the power consumption of a purely digital circuit, which is given by the number of gates and flip-flops, the clock frequency, the supply voltage value or the activity factor. Even though the MPPT contribution to the total power consumption of the chip may be negligible, the target is to send power to the load or batteries, not to cause an extensive energy self-consumption.

Occupied chip area is the last analyzed parameter. The EH system usually consists of digital and, in particular, analog circuits, which take up the largest area and are more difficult to implement in terms of layout. Based on the size of the digital circuit area, we determine not only whether it will fit on the chip at all, but also whether it will fit in the specific location defined by the layout of the analog parts.

The convergence speed and error were obtained by measurement, and the power consumption and occupied area were obtained using tools of the Cadence design environment. Based on the data obtained, it can be concluded that in terms of speed and step size, the Constant Voltage MPPT algorithm consistently achieved the best results. As for the error size, this parameter is mainly influenced by the tuning step size and the amount of incident radiation on the solar cells.

Since neither energy consumption nor occupied area depend on the tuning step size or the amount of energy obtained, it can be clearly stated that the Pilot-Cell MPPT algorithm achieves the best results in terms of energy consumption. Its consumption is more than 171 times lower than in the worst case with the P&O algorithm. This may be due to the fact that the PC algorithm works with slowly changing 1-bit input information, which leads to a lower value of the activity factor contributing to dynamic consumption.

The smallest chip area among the used MPPT algorithms is that of the Constant Voltage algorithm. Its area is 2.5 times smaller than in the worst case with the P&O algorithm. Using the CV algorithm, we obtain data on the input voltage from the ADC and compare it with a constant. In the case of the P&O algorithm, its decision-making depends on information about the input power and voltage. The necessary power data is not obtained directly by measurement, but is derived by multiplying the input voltage and current values obtained from the ADC. The circuit that performs the multiplication of two numbers is not simple, and this is where the largest increase in chip area occurs. All the results achieved confirm the assumptions made in the theory.

**Figure 21:**
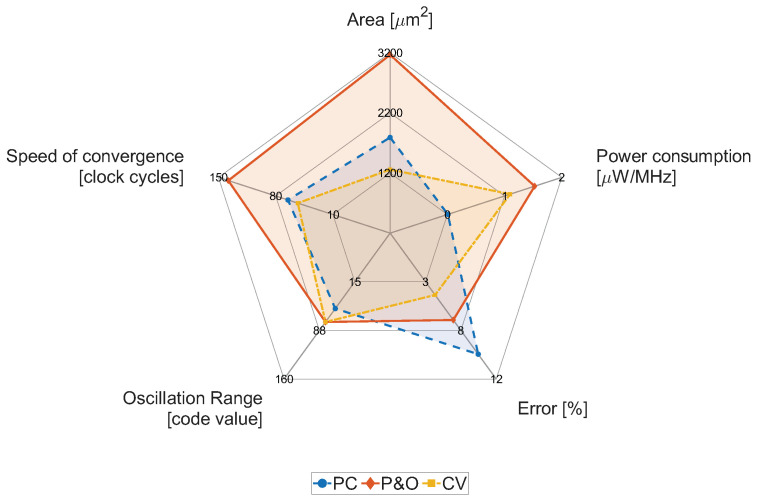
Representative results of key properties of each inspected MPPT algorithm among 10 chip samples.

The proposed energy harvester represents the first iteration of our research in the field of development and optimization of solar-based energy harvesting on a chip, which was built on theoretical and simulation knowledge. The aim of this work was to investigate the influence of individual MPPT algorithms on selected parameters of the energy harvester system. In Figure 21, the radar chart comparing applied and evaluated MPPT algorithms in terms of main parameters is shown.

The Pilot-Cell algorithm dominates with the smallest range of oscillations around the MPP (936-921 in register code) and also especially in energy consumption (dynamic + static), which is only 8.9nW/1MHz. The P&O algorithm has comparable properties in terms of oscillation range and error rates with other algorithms. Still, it lags far behind in the different parameters (area, power consumption, and speed of convergence). The last tested CV algorithm is essentially the PC algorithm, but the decision-making occurs based on data from the ADC rather than from a voltage comparator. Such a circuit achieves the best results in terms of convergence speed, requiring only 26 clock cycles (and in the worst case, 53 clock cycles). It occupies the smallest area of $1258\;\mu m^{2}$, and its error rate ranges from 3.05% to 11.52%. The performance of the proposed MPPT algorithms is summarized in Table 5. A comparison is made with state-of-the-art EH systems employing PV (or hybrid PV-based) power sources and MPPT algorithms. All compared solutions are manufactured using either 65 nm or 180 nm CMOS technology. The achieved MPPT efficiency $\eta_{MPPT}$ (obtained as 100% − ErrMPPT), is comparable to that reported in related works. The main advantages of on-chip implemented MPPT architectures include low power consumption and a compact footprint. However, the overall energy conversion efficiency and the maximum output power are lower. This reduction can be attributed primarily to the full integration of the inductor on the chip, which limits the achievable current throughput of the system. Specifically, the integrated inductor used in this work exhibits an inductance of 11.6 nH, while state-of-the-art reference designs rely on discrete inductors with significantly higher inductance values, ranging from 4.7 μH [44] to 4.7 mH [45]. The limitations of the power converter do not influence the performance of the employed MPPT algorithms.

## 9. Conclusions

We have designed an energy harvester ASIC intended for low-power electronics that was prototyped using 65 nm CMOS technology by UMC. Most of the system components, such as the DC-DC converter, MPPT controller, relaxation oscillator, and one-shot oscillator, have been fully integrated on the chip. Other components such as a shunt regulator, evaluation and measurement circuits (i.e., a voltage comparator and ADC), and PV cells were used in discrete form. Different algorithms were implemented in the MPPT controller and their properties were compared to find the most proper solution for increasing the overall EH efficiency. Indirect MPPT algorithms require information about the voltage of the loaded solar cell ($V_{PV}$) and the voltage of the unloaded solar cell ($V_{OV}$). In practice, this means that to obtain the necessary $V_{OV}$, it is essential to disconnect the solar cells from the rest of the circuit, preventing energy transfer for a brief moment. To avoid interrupting the supply of converted energy, we installed another identical pair of solar cells next to the existing one. This configuration provides $V_{PV}$ from one pair of solar cells and $V_{MPP}$ from the other. To manage the energy extraction from the aforementioned solar cell assembly, the Pilot-Cell MPPT algorithm was used. Its selected features can be configured using registers controllable via a computer. It is possible to choose whether the algorithm will operate with an adaptive or static step. In the case of a static step, the size of the convergence step, ranging from 1 to 31, can be selected.

From performed measurements of ASIC samples and results obtained by applying individual MPPT algorithms, we evaluated the main parameters such as area, power consumption, error from the MPP, oscillation range and speed of convergence. Based on these data, a comparison of MPPT algorithms was made.

Firstly, the Pilot-Cell algorithm was analyzed. It processes 1-bit information from the comparator. In terms of energy consumption and oscillation range, it achieves the lowest values among the others, thus dominating in two key parameters out of five. The second analyzed algorithm was Constant Voltage, which is similar to PC in its operating principle. However, it works with multi-bit information from the ADC. Attributes such as power consumption and oscillation range acquire higher values than PC. Power consumption may be caused by higher circuit complexity due to ADC data capture, and an increase in oscillation range may be caused by an incorrectly selected value of a constant representing $V_{mpp}$. Error, convergence speed, and area achieve lower values. This means that it dominates in three out of five evaluated aspects. The last investigated algorithm is P&O. It is classified as a direct algorithm and uses voltage and current information from the ADC. This type of algorithm achieves the worst results in power consumption, size of area and convergence speed. The reason why the power consumption and area achieved the worst results compared to other algorithms was mentioned in the Section 8. However, the convergence speed is affected by the capture, multiplication, and evaluation of data such as input power and voltage, which takes several clock cycles, and the tuning step changes only by a constant value. As for the error of the MPP and oscillation range, the achieved results are comparable to other MPPT algorithms.

According to the analysis described above, the CV algorithm achieves the best results among the evaluated MPPT algorithms. The disadvantage is energy consumption, and reducing it will be one of the goals of future work. The PC algorithm also achieves excellent results, but despite processing 1-bit information from the comparator, it requires more chip area than CV. In this regard, it will also be necessary to perform optimization steps according to the area. The most complex analyzed algorithm, P&O, lags behind in several parameters. Despite its less favorable characteristics, we see great potential for future improvements. Efforts will be directed towards approximating the characteristics of indirect algorithms as closely as possible, especially in terms of convergence speed and energy consumption.

## Figures and Tables

**Table 1:** Comparison of selected energy converters in terms of main parameters.

	Photovoltaic	Thermoelectric	Piezoelectric	RF	Biofuel Cell
**Output voltage**	≈600 mV	<100 mV	<150 V	1–4 V	<300 mV
**Output power**	100 $\mathrm{mW}/{\mathrm{cm}}^{2}$	50–100 $\mu W/{\mathrm{cm}}^{2}$	15 W	<1 W	12 $\mu W/{\mathrm{cm}}^{2}$
**Efficiency**	15–25%	1–17%	50–90%	1–90%	<12%

**Table 2:** Comparison of selected indirect and direct MPPT algorithms under uniform conditions.

Algorithm	PV Cell Dependency	Sensor		Tracking Speed	Efficiency
		V	I		
Constant Voltage	YES	✓		FAST	LOW
Open Circuit Voltage	YES	✓		FAST	LOW
Short Circuit Current	YES		✓	FAST	LOW
Pilot-Cell	YES	✓		FAST	LOW
Perturb & Observe	NO	✓	✓	SLOW	HIGH
Incremental Conductance	NO	✓	✓	MEDIUM	HIGH

**Table 3:** Achieved results of the EH using different MPPT algorithms under irradiation $425\;W/m^{2}$ (for tuning step values of 15 and 31).

Algorithm	PC		P&O		CV	
Step	15	31	15	31	15	31
**Speed of Convergence** [clock cycles]	57	38	146	48	49	26
**Error of $V_{IN}$ from $V_{MPP}$** [%]	6.19	7.36	6.03	8.39	5.02	11.09
**Power Consumption** [μW/1MHz]	0.0089		1.5262		1.0976	
**Area** [$\mu m^{2}$]	1791		3166		1258	

**Table 4:** Achieved results of the EH using different MPPT algorithms under irradiation $500\;W/m^{2}$ (for tuning step 15 and 31).

Algorithm	PC		P&O		CV	
**Step**	**15**	**31**	**15**	**31**	**15**	**31**
**Speed of Convergence** [clock cycles]	65	41	138	54	53	27
**Error of $V_{IN}$ from $V_{MPP}$** [%]	9.73	11.52	6.54	9.58	4.22	3.05
**Power Consumption** [μW/1MHz]	0.0089		1.5262		1.0976	
**Area** [$\mu m^{2}$]	1791		3166		1258	

**Table 5:** Performance of analyzed MPPT algorithms in designed EH compared to State-of-the-Art energy harvesting systems with MPPT.

	Energy Source (V_*in*_) [V]	${P_{\mathrm{out}}}_{\max}$ [μW]	$\eta_{E2E}$ [%]	$\eta_{\mathrm{MPPT}}$ [%]	MPPT (Area [mm^2^])	Power Consumption [μW/MHz]
Our Work ^1^	PV (<1.38)	500	30–43.6	80.6–97.2	PC (0.00179)	0.0089 ^3^
					P&O (0.00317)	1.5262 ^3^
					CV (0.00126)	1.0976 ^3^
[46] ^2^ 2025	PZT	110	88 (PZT),	95–99.3	FOCV (0.019 ^4^)	0.0105
	PV		85.7 (PV)			Asynchronous
[47] ^2^ 2023	PZT (1–1.6)	2269	86.67	99.58 (PZT)	FS, FOCV	-
	TEG (0.05–0.65)			99.37 (TEG)	(0.249 ^4^)	
[48] ^1^ 2022	TEG	1200	-	66–81.5 (PV)	FOCV	-
	PV					
	PZT					
[44] ^2^ 2021	PV (1.45–1.8)	24,000	90.2	98.9	OCV (0.159)	174.96
	BFC (0.2–0.7)					including
	TEG (0.03–0.09)					comparators
[45] ^2^ 2020	PV (1.8)	10,000	86	97.2	FOCV	-
[49] ^2,3^ 2020	PV (0.32–0.425)	500	68.3	92	Time-Based	-

^1^ 65 nm CMOS, ^2^ 180 nm CMOS, ^3^ Simulation results, ^4^ Estimated from Chip Photograph.

## Data Availability

No new datasets were created in this research.
